# Factors Associated with Dissatisfaction with Out-of-Pocket Medical Costs Among Medicare Beneficiaries

**DOI:** 10.1093/geroni/igaf122.4116

**Published:** 2025-12-31

**Authors:** Andrew Chern, Boon Peng Ng

**Affiliations:** University of Central Florida, Orlando, Florida, United States; University of Central Florida, Orladno, Florida, United States

## Abstract

Out-of-pocket medical expenses remain a substantial concern for many Medicare beneficiaries, particularly those with high care needs and limited income. The objective of this study was to examine factors (i.e., sociodemographic characteristics, health status, insurance coverage and benefits) associated with dissatisfaction with out-of-pocket medical costs among Medicare beneficiaries. We conducted a cross-sectional analysis of 9,960 adults aged 65 years and older from the 2022 Medicare Current Beneficiary Survey Public Use File (weighted N = 51.7million). The dependent variable was self-reported satisfaction with out-of-pocket costs, dichotomized as satisfied versus dissatisfied. A survey-weighted logistic regression model identified factors associated with dissatisfaction. Overall, 12.4% (6.4million) reported dissatisfaction. Higher odds of dissatisfaction were associated with age 65–74 years versus ≥75 (OR = 1.41,P< 0.001), non-Hispanic Black (OR = 1.44,P=0.011) or other race/ethnicity (vs non-Hispanic White), income ≤120% federal poverty level (FPL) (OR = 1.46,P=0.023) or > 120% to ≤ 200% FPL (vs > 200% FPL), 1–2 limitations in activities of daily living (ADLs) (OR = 1.60,P< 0.001) or ≥ 3 limitations (vs no functional limitations), reporting good general health (OR = 1.31,P=0.001) or fair/poor health (vs excellent/very good), having a Part D plan (vs no) (OR = 1.29,P=0.033), and self-purchased private insurance (vs no) (OR = 1.30,P=0.013). Full-year Medicare Advantage enrollment (vs. full-year fee-for-service) (OR = 0.62,P< 0.001) and dual Medicare–Medicaid eligibility (vs no dual) (OR = 0.34,P< 0.001) were protective factors against dissatisfaction. These findings highlight inequities in out-of-pocket costs satisfaction among socioeconomically and medically vulnerable older adults. Policy reforms aimed at expanding supplemental coverage and providing targeted financial assistance could improve out-of-pocket cost-related satisfaction and advance health equity in this population.

